# Nonlethal Plasmodium yoelii Infection Drives Complex Patterns of Th2-Type Host Immunity and Mast Cell-Dependent Bacteremia

**DOI:** 10.1128/IAI.00427-20

**Published:** 2020-11-16

**Authors:** Nora Céspedes, Erinn Donnelly, Sarah Garrison, Lori Haapanen, Judy Van De Water, Shirley Luckhart

**Affiliations:** a Department of Entomology, Plant Pathology, and Nematology, University of Idaho, Moscow, Idaho, USA; b Department of Biological Sciences, University of Idaho, Moscow, Idaho, USA; c Division of Rheumatology, Allergy, and Clinical Immunology, University of California, Davis, California, USA; UC Davis School of Veterinary Medicine

**Keywords:** malaria, allergy, bacteremia, mast cells, cytokines, *Plasmodium yoelii*

## Abstract

Malaria strongly predisposes to bacteremia, which is associated with sequestration of parasitized red blood cells and increased gastrointestinal permeability. The mechanisms underlying this disruption are poorly understood. Here, we evaluated the expression of factors associated with mast cell activation and malaria-associated bacteremia in a rodent model. C57BL/6J mice were infected with Plasmodium yoelii *yoelli* 17XNL, and blood and tissues were collected over time to assay for circulating levels of bacterial 16S DNA, IgE, mast cell protease 1 (Mcpt-1) and Mcpt-4, Th1 and Th2 cytokines, and patterns of ileal mastocytosis and intestinal permeability. The anti-inflammatory cytokines (interleukin-4 [IL-4], IL-6, and IL-10) and MCP-1/CCL2 were detected early after P. yoelii *yoelii* 17XNL infection. This was followed by the appearance of IL-9 and IL-13, cytokines known for their roles in mast cell activation and growth-enhancing activity as well as IgE production. Later increases in circulating IgE, which can induce mast cell degranulation, as well as Mcpt-1 and Mcpt-4, were observed concurrently with bacteremia and increased intestinal permeability. These results suggest that P. yoelii *yoelii* 17XNL infection induces the production of early cytokines that activate mast cells and drive IgE production, followed by elevated IgE, IL-9, and IL-13 that maintain and enhance mast cell activation while disrupting the protease/antiprotease balance in the intestine, contributing to epithelial damage and increased permeability.

## Abstract (precis)

Malaria strongly predisposes to bacteremia, which is associated with sequestration of parasitized red blood cells and increased gastrointestinal permeability. The mechanisms underlying this disruption are poorly understood. Here, we evaluated the expression of factors associated with mast cell activation and malaria-associated bacteremia in a rodent model. C57BL/6J mice were infected with Plasmodium yoelii *yoelli* 17XNL, and blood and tissues were collected over time to assay for circulating levels of bacterial 16S DNA, IgE, mast cell protease 1 (Mcpt-1) and Mcpt-4, Th1 and Th2 cytokines, and patterns of ileal mastocytosis and intestinal permeability.

## INTRODUCTION

According to the World Health Organization (WHO), there were an estimated 228 million cases of malaria and 405,000 deaths worldwide in 2018. Most cases occur in sub-Saharan Africa (93%) and are due to infection with Plasmodium falciparum. In African children diagnosed with severe falciparum malaria, approximately 5% develop invasive bloodstream infections or bacteremia with a higher case fatality (24.1%) relative to children with malaria alone (1). While the connection between severe malaria and bacteremia is clear (2–4), there is growing evidence to suggest that bacteremia is associated with a broader spectrum of malarial disease than was previously appreciated. Notably, a prospective 2018 study in Ghana revealed an inverse correlation between the likelihood of nonmalarial coinfections, including bacteremia, with increasing circulating parasitemia, suggesting that undetected bacteremia in asymptomatic, parasitemic children could pose significant risk of death without intervention (5). Other studies have reported an association between Gram-negative bacteremia and reduced malaria parasitemia along with increased susceptibility to severe malarial anemia and respiratory distress in children (2, 4). In contrast to previous assumptions that bacteremia is uncommon in adults with malaria, bacteremia was detected more frequently (15%) in hospitalized adults with falciparum malaria from Myanmar than previously reported; the standard practice of empirical antibiotic treatment in these combined studies at admission was likely cause and effect with high patient survivorship (6, 7).

The physiology that predisposes to bacteremia in malaria is expected to be largely the same in both children and adults (6). That is, the sequestration of P. falciparum-infected red blood cells (RBCs) results in intestinal capillary blockage (8), malabsorption, and increased gastrointestinal permeability in both acute and chronic infection (9–11). If we assume this argument is correct, the occurrence of bacteremia across a broad clinical spectrum of falciparum malaria would suggest that divergent patterns of host immunity to infection—ranging from severe malaria with a proinflammatory or T helper type 1 (Th1)-skewed response to a combination of antiparasite and antidisease immunity associated with higher levels of anti-inflammatory or Th2-type cytokines with increasing age and exposure (12–15)—also include shared host responses across the clinical spectrum of malaria that precipitate bacteremia. We propose that a potential common mediator of intestinal permeability across the spectrum of malarial disease is intestinal mastocytosis.

Previous studies with our mouse malaria models established that mast cell (MC) activation is functionally involved in increased intestinal permeability and bacteremia. Specifically, we observed that Plasmodium yoelii-infected mice develop l-arginine deficiency, which we associated with intestinal mastocytosis, elevated ileal and plasma histamine, and enhanced intestinal permeability and bacteremia that were reversed with l-arginine supplementation (16). In addition, we used MC-deficient WBB6F1/J-*Kit^W^*/*Kit^W-v^* mutant mice and wild-type littermate controls to establish that MC deficiency is associated with significantly reduced malaria-induced gastrointestinal permeability, improved adherent junction integrity in the ileal epithelium, and reduced enteric bacterial translocation to the spleen, liver, and blood (17). We also showed that antihistamine treatment of P. yoelii-infected mice resulted in partial reversal of bacteremia compared to infected control mice (18). These observations are consistent with clinical findings of l-arginine deficiency in falciparum malaria (19, 20), elevated plasma histamine (21–23), and activation of basophils (24–26), which along with MCs are the principle sources of histamine release during allergic inflammation.

The physiology of MC activation is complex, involving numerous potential mediators that act in response to distinct stimuli over broad time intervals. Notably, MCs respond to microorganisms through a wide variety of membrane receptors (27, 28), as well as to cytokines, chemokines, and other inflammatory signals (28). Cytokines and chemokines typically associated with MC activation include stem cell factor (SCF), the Th2 cytokines interleukin-3 (IL-3), IL-4, IL-5, IL-6, IL-9, and IL-13, granulocyte-macrophage colony-stimulating factor (GM-CSF) (17, 29, 30), and regulated on activation normal T cell expressed and secreted (RANTES CCL5) (31) and eotaxin (CCL11) (32). The Th2 cytokine IL-10 can induce MC proliferation in combination with SCF and IL-6 (33). Together, IL-4 and IL-10 can inhibit some MC functions while inducing others (34). The most clinically relevant example of MC activation is associated with type I hypersensitivity, which is mediated by cross-linking of antigen-specific immunoglobulin E (IgE) immune complexes and high-affinity IgE receptors (FcεRI) on the MC membrane surface (27). MCs activated after FcεRI binding undergo degranulation and release of inflammatory mediators such as histamine, heparin, proteoglycans, Th1 and Th2 cytokines, and proteases, including some that can directly degrade circulating cytokines (35) to modulate the recruitment, survival, proliferation, and activation of other leukocytes (36, 37).

Here, we evaluated a timeline of MC activation in a nonlethal malaria model along with patterns of peripheral leukocytes and circulating pro- and anti-inflammatory cytokines and chemokines to improve our understanding of bacteremia in the recently clinically relevant context of nonsevere disease. The C57BL/6J mouse model of infection with nonlethal P. yoelii *yoelii* 17XNL resembles protective immunity and tolerance to P. falciparum, which is defined by both Th1 and Th2 responses. These responses have been described as contributing to antiparasite immunity (Th1) and antidisease immunity (Th2), but the roles of Th2 responses during blood-stage infection, other than to promote IL-4-dependent protective humoral immunity (38) and reduce macrophage antiparasite responses (39), remain incompletely understood. In the present study, our observations define a Th2-type allergic response during nonlethal malaria marked by transient increases in basophils, eosinophils, and neutrophils together with lymphopenia, ileal mastocytosis, and elevated levels of Th2 cytokines and chemokines, providing unique temporal patterns for interpreting MC-dependent, malaria-induced alterations to the intestinal barrier.

## RESULTS

### Nonlethal P. yoelii *yoelii* 17XNL infection was associated with rising bacteremia within 2 days of detectable parasitemia.

**FIG 1:**
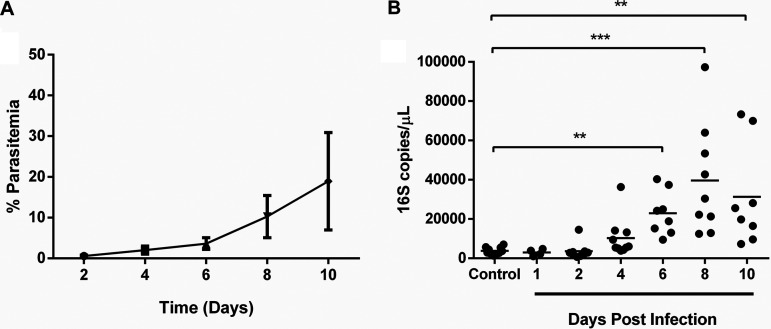
(A and B) Peripheral blood parasitemia following P. yoelii *yoelii* 17XNL infection (A) and bacterial 16S copies/μl of blood (B) in C57BL/6J mice relative to control, uninfected mice. The error bar (A) represents the mean ± standard deviation (SD). Each dot (B) represents a single mouse. Data (B) were analyzed with the Kruskal-Wallis test followed by Dunn’s multiple comparison of each time point with the control group. *P* values of <0.05 were considered significant. **, *P* ≤ 0.01; ***, *P* ≤ 0.001.

To define the temporal association between malaria-associated bacteremia and MC activation in our model of nonlethal infection, wild-type C57BL/6J mice were injected intraperitoneally with P. yoelii *yoelii* 17XNL-infected red blood cells. Intestinal permeability and MC activation and accumulation were assessed during early infection to approximately peak parasitemia (10 days postinfection [p.i.]) (18). All mice injected with infected red blood cells were positive for infection by 2 days postinoculation (Fig. 1A). Increased bacterial 16S DNA levels in blood were observed by day 4 p.i. (mean parasitemia at day 4 p.i., 2.0%) and became significantly different from controls by day 6 p.i. (Fig. 1B).

**FIG 2:**
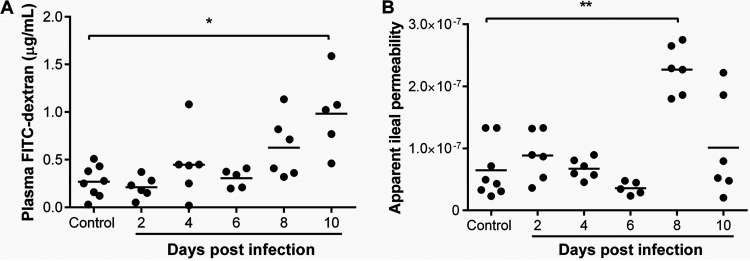
Intestinal permeability during P. yoelii *yoelii* 17XNL infection. (A) Intestinal permeability *in vivo* quantitated with FITC-dextran in plasma of infected and control, uninfected mice after administration of FITC-dextran by oral gavage. (B) *Ex vivo* intestinal permeability determined from FITC-dextran passage across ligated, resected ileum sacs. Each dot represents a single mouse. Data were analyzed with the Kruskal-Wallis test followed by Dunn’s multiple comparison of each time point with the control group. *P* values of <0.05 were considered significant. *, *P* ≤ 0.05; **, *P* ≤ 0.01.

Gastrointestinal (GI) permeability was determined using *in vivo* and *ex vivo* assays in P. yoelii *yoelii* 17XNL-infected and control, uninfected mice. Permeability was assessed *in vivo* by measuring the plasma concentration of 4-kDa fluorescein isothiocyanate dextran (FITC-dextran) at 3 h following oral gavage of mice. With increasing intestinal barrier damage and paracellular permeability, greater densities of FITC-dextran particles are detected in the plasma. Using this assay, increased intestinal permeability was observed by day 4 p.i. that became significantly different from uninfected controls by day 10 p.i. (Fig. 2A). Plasma FITC-dextran levels were significantly but moderately correlated with circulating bacterial 16S (*P* = 0.010, *r* = 0.589; see Fig. S1 in the supplemental material), suggesting that a substantial amount of variation in permeability was unaccounted for by this assay. As a secondary assay, permeability was measured *ex vivo* by transport of 4-kDa FITC-dextran across resected, ligated sections of ileum from infected and control mice, allowing direct and localized assessment of GI barrier integrity (40). In this assay, relative permeability is calculated as a function of width and length of the intestinal segment and FITC-dextran released from the “ileum sac” into the suspension medium over time. In contrast to the *in vivo* assay, the *ex vivo* test revealed significantly increased ileal permeability by day 8 p.i. that declined by day 10 p.i. (Fig. 2B).

**FIG 3:**
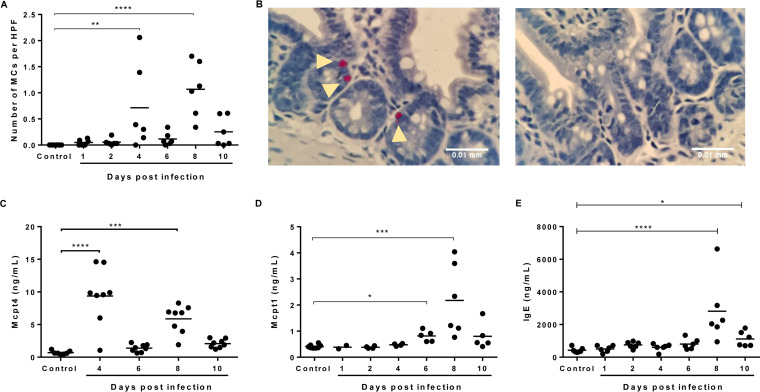
Association of P. yoelii *yoelii* 17XNL infection with accumulation and activation of ileal mast cells (MCs). (A) Mean numbers of ileal MCs per high-powered field (HPF) from naphthol AS-D chloroacetate esterase (NASDCE) staining of sections from infected and control, uninfected mice. (B) Representative stained MCs (pink cells indicated by white arrows) in the ileum of an infected mouse at 8 days p.i. (left) and a control mouse (right). (C) MC protease 4 (Mcpt-4) concentration in plasma as determined by ELISA. (D) MC protease 1 (Mcpt-1) concentration in plasma as determined by ELISA. (E) IgE concentration in plasma as determined by ELISA. Data were analyzed with the Kruskal-Wallis test follows by Dunn’s multiple comparison of each time point with the control group. *P* values of <0.05 were considered significant. *, *P* ≤ 0.05; **, *P* ≤ 0.01; ***, *P* ≤ 0.001; ****, *P* ≤ 0.0001.

In the context of rising bacteremia by day 4 p.i. that was significant by day 6 p.i. (Fig. 1B), significantly increased numbers of MCs, identified using naphthol AS-D chloroacetate esterase (NASDCE) activity, which specifically detects MC secretory granule chymases, were observed in the ileum by days 4 and 8 p.i. relative to controls (Fig. 3A and B). MC activation, as interpreted by elevated levels of circulating MC protease 4 (Mcpt4) and Mcpt1, was significant by days 4 and 8 and by days 6 and 8, respectively (Fig. 3C and D). Elevated levels of IgE were observed at 8 days p.i. (Fig. 3E). The functional human chymase homologue Mcpt4 has been associated with increased intestinal permeability (35, 41, 42), decreased infection-induced intestinal inflammation, and regulation of intestinal cytokine responses (43). Mcpt1 release is significantly upregulated by allergen-dependent IgE cross-linking (44) and, like Mcpt4, has been associated with increased intestinal permeability (45), suggesting that MC activation at day 4 p.i. initiates an uptick in circulating bacterial 16S levels that were significantly increased above control levels by day 6 p.i. (Fig. 1B) by the combined activities of Mcpt1 and Mcpt4. By day 10 p.i., ileal MC numbers (Fig. 3A) and activation (Fig. 3C and D) were not different from those of controls, but circulating 16S levels in infected mice remained significantly higher than those of controls (Fig. 1B).

### Patterns of circulating leukocytes during P. yoelii *yoelii* 17XNL infection are reminiscent of allergic inflammation and consistent with nonlethal disease.

**FIG 4:**
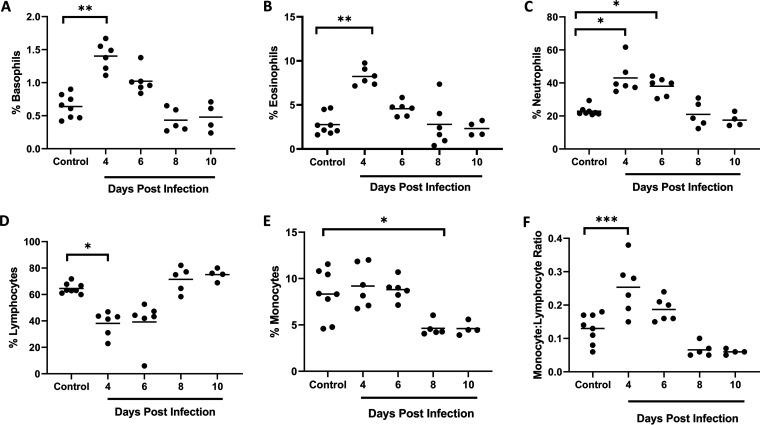
Circulating leukocytes in P. yoelii *yoelii* 17XNL-infected and control, uninfected mice. (A to F) The *x* axis represents the time points in days after infection, and the *y* axis represents the percentages of circulating basophils (A), eosinophils (B), neutrophils (C), lymphocytes (D), and monocytes (E) and monocyte/lymphocyte ratios (F). Each dot represents a single mouse. Data were analyzed with the Kruskal-Wallis test followed by Dunn’s multiple comparison of each time point with the control group. *P* values of <0.05 were considered significant. *, *P* ≤ 0.05; **, *P* ≤ 0.01; ***, *P* ≤ 0.001.

To determine the profile of immune cells in our nonlethal malaria model in the context of bacteremia, we quantified circulating basophils, eosinophils, neutrophils, lymphocytes, and monocytes in P. yoelii *yoelii* 17XNL-infected and control, uninfected mice. Following infection, significant increases in basophils and eosinophils were noted by day 4 p.i. (Fig. 4A and B), the same day that elevated MCs were first observed in the ileum (Fig. 3A), with both basophils and eosinophils declining to control levels by day 8 p.i. Basophils are rare c-kit receptor-negative, FcεRI-positive cells that mature in the bone marrow and that, upon activation, release histamine, lipid mediators, and chemokines, as well as IL-4 and IL-13 (46, 47). Basophils are key players in Th2 immune responses (48), which are characterized by activation and recruitment of basophils, MCs, and eosinophils (49) and the production of IL-4, -5, -6, -9, -10, and -13, which promote secretion of IgE to sustain MC activation (46, 47, 50).

Interestingly, circulating neutrophils followed a pattern of increase and decline relative to controls (Fig. 4C) that was similar to basophils and eosinophils. In the context of IgE-mediated allergy, activated MCs have been reported to induce an increase in infiltrating neutrophils (51), which respond to local cytokines by presenting antigen to specific CD4^+^ effector T-cells (52) that release IL-5 to activate eosinophils and increase the synthesis of IgE (53). Lymphocytes, in contrast, were significantly decreased in circulation by day 4 p.i. and returned to baseline by day 8 p.i. (Fig. 4D), whereas monocytes remained at control levels through day 6 p.i. and then declined thereafter (Fig. 4E). The monocyte to lymphocyte ratio has been used as a marker of infection in a range of diseases (54–56), including malaria, where positive correlations with parasitemia and severe disease have been noted (57, 58). Relative to controls, monocyte to lymphocyte ratios were significantly increased by day 4 p.i. and then declined by day 6 p.i. with values below baseline at days 8 and 10 p.i. (Fig. 3F), suggesting a transient increase and then decline in disease severity with increasing parasitemia as expected for a nonlethal infection. This transition may also reflect a shift from innate to adaptive immunity, marked for example, by notably increased synthesis of IgE by day 8 p.i. (Fig. 3E). Concurrent transient eosinophilia and lymphopenia, together with elevated levels of Th2 cytokines, have been described in murine experimental asthma (59), suggesting that the patterns observed here define a variant Th2-type allergic response that is associated with early ileal mastocytosis, MC activation, and bacteremia.

### Patterns of plasma cytokines and chemokines during P. yoelii *yoelii* 17XNL infection are consistent with MC activation and both antiparasite and antidisease immunity.

In the lethal malaria model of Plasmodium berghei infection, Th2-associated allergic mediators, including histamine, have been associated with increased parasitemia (60) and increased severity of cerebral disease during infection (61). Conversely, we have observed in the nonlethal model of P. yoelii *yoelii* 17XNL infection that histamine is associated with decreased parasitemia and with pathology marked by ileal mastocytosis and intestinal epithelial cell damage that are functionally associated with enteric bacterial translocation (18). Given that asymptomatic infection and tolerance in falciparum malaria have been associated with bacteremia (5–7) and based on our observations of pathology that were reminiscent of allergic inflammation (Fig. 3 and 4), we examined the patterns of Th1 and Th2 cytokines and chemokines in our model.

**FIG 5:**
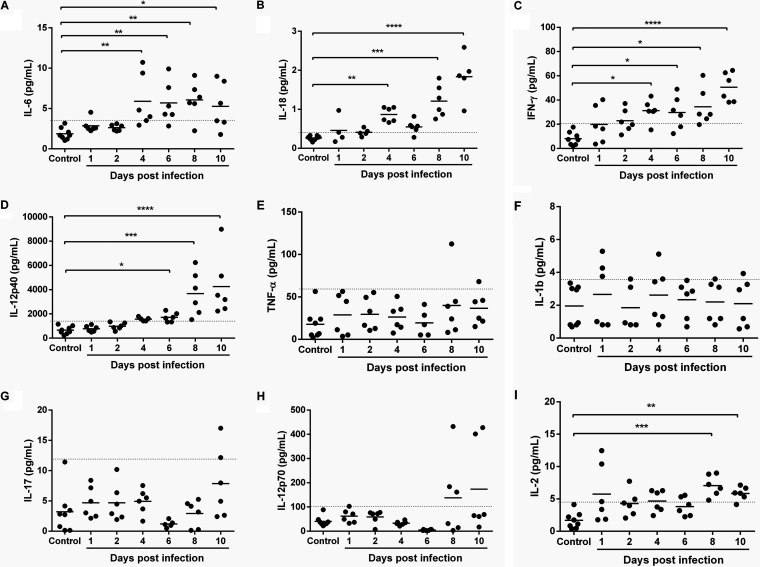
Proinflammatory cytokines in plasma of P. yoelii *yoelii* 17XNL-infected and control mice. (A to I) The *x* axis represents the time points in days after infection, and the *y* axis represents the plasma concentrations of IL-6 (A), IL-18 (B), IFN-γ (C), IL-12p40 (D), TNF-α (E), IL-1β (F), IL-17 (G), IL-12p70 (H), and IL-2 (I). Each dot represents a single mouse. Data were analyzed with the Kruskal-Wallis test followed by Dunn’s multiple comparison of each time point with the control group. *P* values of <0.05 were considered significant. *, *P* ≤ 0.05; **, *P* ≤ 0.01; ***, *P* ≤ 0.001; ****, *P* ≤ 0.0001.

**FIG 6:**
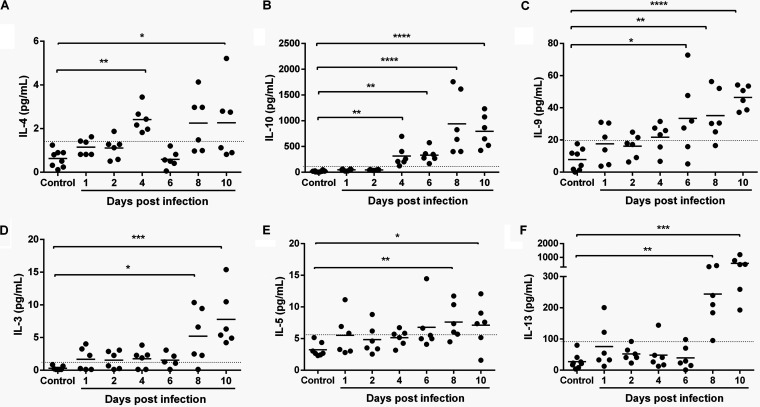
Anti-inflammatory cytokines in plasma of P. yoelii *yoelii* 17XNL-infected and control, uninfected mice. (A to F) The *x* axis represents the time points in days after infection, and the *y* axis represents the plasma concentrations of IL-4 (A), IL-10 (B), IL-9 (C), IL-3 (D), IL-5 (E), and IL-13 (F). Each dot represents a single mouse. Data were analyzed with the Kruskal-Wallis test followed by Dunn’s multiple comparison of each time point with the control group. *P* values of <0.05 were considered significant. *, *P* ≤ 0.05; **, *P* ≤ 0.01; ***, *P* ≤ 0.001; ****, *P* ≤ 0.0001.

In general, patterns of circulating cytokines over time following P. yoelii *yoelii* 17XNL infection showed evidence of dual activation and cross talk between Th1 and Th2 responses, with the earliest increases (day 4 p.i.) in IL-6, IL-18, and interferon-γ (IFN-γ) (Fig. 5A to C) along with IL-4 and IL-10 (Fig. 6A, B), which corresponded to the first peak of intestinal mastocytosis (Fig. 3A). The early appearance of IL-4, in the absence of IL-13, highlights important differences in these two cytokines that share a signaling receptor subunit IL-4 receptor-α (62). Specifically, IL-4 has been identified as the first cytokine to be produced by MCs and is responsible for promoting MC IL-13 production (63). In addition, IL-4 functions as a key amplifier of Th2 immunity (64). In mouse malaria, increased levels of IL-10 have been associated with development of nontyphoidal *Salmonella* (NTS) bacteremia, suggesting that IL-10 suppresses mucosal inflammatory responses to invasive NTS (65). Increased levels of IL-18 likely balance the amplification of Th2 immunity. A primary function of IL-18 is to induce the synthesis of IFN-γ (Fig. 5C), an outcome that is absent in some acute allergic conditions (66). Here, IL-18-dependent Th1 immunity and the likely engagement of IFN-γ in blood-stage parasite killing (67) appear to be sustained for the duration of rising P. yoelii *yoelii* 17XNL parasitemia. However, IL-18 can also participate directly in the proliferation and recruitment of MCs and basophils observed by day 4 p.i. (Fig. 3A and 4A), it contributes to the synthesis of IL-4 and IL-13 in a variety of innate immune cells, including NK cells, MCs, and basophils, and in the presence of allergen, it can increase the synthesis of IgE (68). Collectively, IL-6, IL-18, IFN-γ, and IL-10 levels rose with parasitemia, while significant increases in IL-4 were biphasic, with a second peak occurring at day 10 p.i.

**FIG 7:**
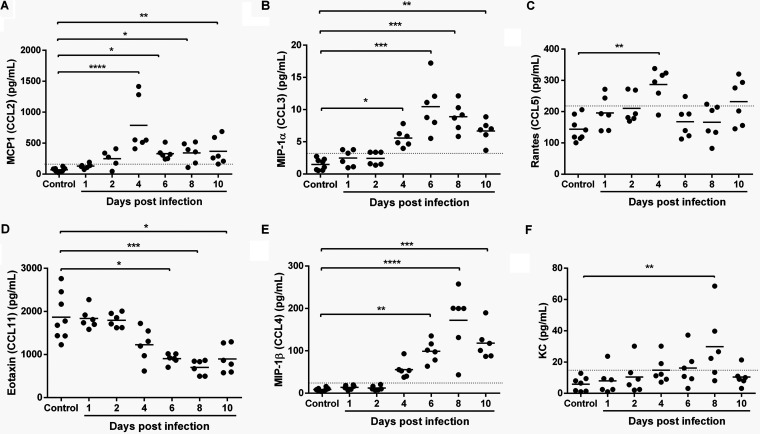
Chemokines in plasma of P. yoelii *yoelii* 17XNL-infected and control, uninfected mice. (A to F) The *x* axis represents the time points in days after infection, and the *y* axis represents the plasma concentrations of MCP-1 (A), MIP-1α (B), RANTES (C), eotaxin (D), MIP-1β (E), and KC (F). Each dot represents a single mouse. Data were analyzed with the Kruskal-Wallis test followed by Dunn’s multiple comparison of each time point with the control group. *P* values of <0.05 were considered significant. *, *P* ≤ 0.05; **, *P* ≤ 0.01; ***, *P* ≤ 0.001; ****, *P* ≤ 0.0001.

The significant increase by day 4 p.i. and rising levels of monocyte chemoattractant protein-1 (MCP-1) (CCL2) (Fig. 7A) and macrophage inflammatory protein-1α (MIP-1α) (CCL3) (Fig. 7B) with parasitemia are notable in that MIP-1α is required for physiologically relevant levels of MC activation *in vivo* (69), and MIP-1α (70) and MCP-1 (71), along with RANTES (Fig. 7C), can induce histamine release by basophils (72). MCP-1 synthesis is also induced by MC activation (73) and is involved in activating the migration of monocytes (74), but this response is naturally antagonized by eotaxin (75), levels of which were sustained through day 4 p.i. but then decreased by day 6 p.i. (Fig. 7D). Interestingly, MIP-1α (CCL3) and MIP-1β (CCL4) (Fig. 7B and E) levels increased through day 6/8 p.i. and then declined, while levels of RANTES gradually increased through day 4 p.i. (Fig. 7C), patterns that recall differences between severe falciparum malaria (high levels of MIP-1α/β and significantly lower baseline levels of RANTES) and mild falciparum malaria (lower levels of MIP-1α/β and significantly higher baseline levels of RANTES) in children (76).

By day 6 p.i., IL-12p40 (Fig. 5D) and IL-9 (Fig. 6C) levels were significantly elevated above those of controls and continued to rise through day 10 p.i. Increasing levels of IL-10 and IL-12p40, an antagonist of IL-12, likely blunt the Th1 response, perhaps explaining IFN-γ levels that increased with parasitemia (Fig. 5C) in the absence of any changes in levels of tumor necrosis factor-α (TNF-α) (Fig. 5E), IL-1β (Fig. 5F), or IL-17 (Fig. 5G) during infection. In our model as in other studies (77), IL-12p40 was produced in large excess (∼30- to 40-fold) over levels of IL-12p70, which were increased by days 8 and 10 p.i. but nonsignificantly (Fig. 5H). In the context of pathogen infection, IL-12p40 has also been observed to regulate macrophage recruitment, a positive role in the host response to infection, while also inhibiting overactive Th1 responses (78). Intriguingly, IL-12p40-deficient mice have shown increased susceptibility to P. berghei ANKA cerebral disease, suggesting in our model, a course of nonlethal disease that results from combined antiparasite and antidisease immunity.

By days 8 to 10 p.i., significant increases in IL-2 (Fig. 5I), IL-3, IL-5, and IL-13 (Fig. 6D to F) were detected, timing that corresponded to significant synthesis of IgE (Fig. 3E) and, therefore, a shift to acquired immunity. This shift is driven primarily by IL-5, which also acts in concert with IL-4, IL-9, IL-13, and eotaxin, all expressed coordinately at this time, to orchestrate and enhance allergic inflammation (53). In both P. yoelii *yoelii* 17XNL infection and in falciparum malaria, IL-2 is necessary for the expansion of CD25Foxp3 CD4^+^ T cells (T regulatory cells, or Tregs) (79, 80) and for activating natural killer (NK) cells, which function in the lysis of infected erythrocytes (79–81). High levels of MC activation and IgE synthesis at day 8 p.i. (Fig. 2A, D) suggest that activated MC-derived IL-2 contributes to Treg expansion (82), which also ultimately contributes to the resolution of both mouse and human allergic inflammation. IL-3 is generated by both CD4^+^ T cells and MCs and is a key mediator of murine MC recruitment (83), differentiation, and mediator release, including histamine, IL-4, IL-6, and IL-13 (84–86), suggesting a mechanism for sustained MC activation. The significant increase in keratinocyte chemoattractant (KC) at 8 days p.i. (Fig. 7F) is consistent with the function of this chemokine. In particular, levels of neutrophil-derived KC are highly correlated with intestinal barrier damage (87), which peaks at 8 days p.i. based on circulating 16S levels (Fig. 1B) and intestinal permeability (Fig. 2B) and is suggestive of a coordinated host response with IL-6 to restore mucosal barrier integrity (88). In previous studies, we observed that infection with P. yoelii blunted the mouse intestinal neutrophilic response to invasive NTS (65). In our current model, which lacks the independent and confounding pathology of invasive NTS, our observations provide a more comprehensive analysis of this malaria-associated barrier defect that enables translocation of enteric bacteria. Collectively, these cytokine and chemokine changes slow the development of malarial immunopathology, but the primary expansion of Tregs can also delay the control of parasitemia (80) during MC-enhanced degradation of the intestinal barrier and associated bacteremia.

## DISCUSSION

**FIG 8:**
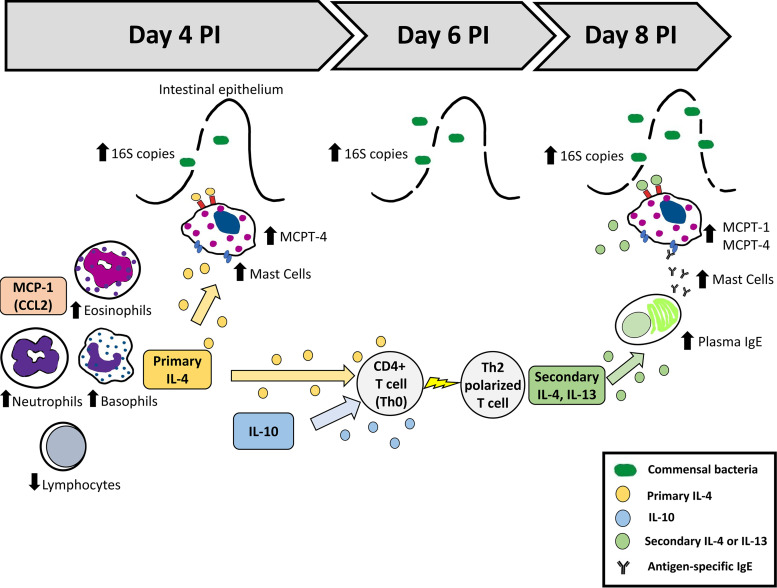
Model for malaria-induced intestinal permeability and bacteremia. Nonlethal malaria (P. yoelii *yoelii* 17XNL) induces transient increases in basophils, eosinophils, and neutrophils together with lymphopenia and synthesis of IL-4, resulting in rapid MC recruitment to the ileum, functionally increased intestinal permeability, and significant elevation of bacterial 16S copies in the blood. These responses are concurrent with a marked Th2-type allergic response and increased MC mediators in circulation.

In this study, we evaluated a timeline of MC activation along with an array of circulating Th1/Th2 mediators to improve our understanding of nonlethal malaria-associated bacteremia and the disruption of intestinal barrier function. In earlier studies, we showed functional associations among ileal mastocytosis, elevated circulating and ileal histamine, enhanced intestinal permeability, and bacteremia in infected mice (16, 18). Here, we characterized early and persistent malaria-induced bacteremia (circulating 16S copies) marked by a Th2-type allergic response and transient increases in basophils, eosinophils, and neutrophils together with lymphopenia, ileal mastocytosis, and elevated circulating levels of MC proteases, Th2 cytokines, and chemokines (Fig. 8). While we have attempted here to interpret changes in the intestinal barrier based, in part, on patterns of circulating immune cells, cytokines, and chemokines, we recognize that these agents are, most importantly, local mediators and that local activities may not be temporally concordant with observed systemic patterns. Nonetheless, these patterns provide for a more comprehensive interpretation of the development of malaria-associated intestinal barrier disruption in the context of nonlethal malaria and are necessary for directing the next steps in our studies of activated, tissue-resident cells and local mediators.

It has been widely accepted that malaria predisposes individuals to bacteremia (3). Gastrointestinal symptoms (89), as well as pathological changes (including detachment of epithelia and shortening of villi and the colon) (90), have been observed during malaria. In severe malaria, infected red blood cells sequester in the gastrointestinal tract (8), causing increased intestinal permeability, rupture, and leakage of infected erythrocytes into the lumen and dysbiosis of the intestinal microbiota, which may contribute to disease severity (90). Bacteremia and intestinal permeability, however, have been observed across all clinical spectra of malaria in both children and adults (1, 3, 5–7). In all scenarios, bacteremia has been frequently associated with reduced parasite densities compared with individuals with malaria only (2, 5). These observations support the relevance of P. yoelii *yoelii* 17XNL infection as a model for the less-well-understood clinical scenario of bacteremia in asymptomatic malaria.

In our model, circulating bacterial 16S DNA copies in blood were increased by 4 days p.i. and were significantly different from controls by 6 days p.i. (Fig. 1B); intestinal permeability to FITC-dextran was significantly increased relative to controls by 8 and 10 days p.i. (Fig. 2A and B). We used two methods to assess the structural permeability of the intestinal barrier, including an *in vivo* method reflective of the entire GI tract and an *ex vivo* method that was specific to resected ileum. Data from these assays were not concordant with each other or with bacteremia. Temporal and regional differences in the permeability of individual intestinal sections could explain these differences. In particular, permeability in the ileum could be higher and more dynamic and exhibit more rapid responses than other intestinal sections (91, 92). These differences could be related to variations in gene expression of tight junction proteins and inflammatory cytokines across intestinal sections (91). Regardless of these differences, barrier permeability that allowed significant passage of FITC-dextran was detected by days 8 to 10 p.i., which in the context of bacteremia and MC activation that precedes and overlaps with these time points, suggests that intestinal barrier disruption occurred at earlier times p.i. but was undetectable with these assays. We are continuing to explore other assays that can more closely associate temporal changes in ileal permeability with bacteremia.

The gastrointestinal mucosa forms a selective barrier that allows the transport of nutrients while protecting the host from potentially harmful pathogens through immune and nonimmune mechanisms. In this context, MCs play a central role in regulating epithelial function and integrity and in modulating both innate and adaptive mucosal immunity (93). MCs produce both proinflammatory and anti-inflammatory cytokines and are an important source of chemokines. As a result, MCs can regulate both Th1 and Th2 responses, depending on the pathogen-associated signal that induces MC activation (94). However, in the intestinal mucosa, MC mediators can also directly affect epithelial integrity, leading to enhanced mucosal permeability and passage of luminal antigens and/or microorganisms across the intestinal epithelium (93).

In addition to its role in regulating MC function, IL-4 has been shown to increase epithelial permeability in various cell types through the induction of pore-forming claudin-2 and apoptotic pathways (95, 96) as well as decreased levels of ZO-1 and occludin (97). IL-13 and IL-6 have also been associated with upregulation of claudin-2 (98–101), which alters cell permeability, lowers transepithelial resistance (TER), and confers increased Na+ conductance (102–104). However, the effects of IL-6 on epithelial permeability remain controversial (105). In our model, both IL-4 and IL-6 were increased in circulation at day 4 p.i. (Fig. 4A and 5A), at the initiation of rising 16S copy numbers in blood (Fig. 1B), suggesting that these cytokines induce early disruption of the intestinal barrier that was enhanced at day 8 p.i. by other mediators, including IL-13, IFN-γ, MC proteases, and histamine.

Among the cytokines measured in our model, TNF-α is among the most studied cytokines that affect intestinal barrier dysfunction. In particular, TNF-α plays a critical role in inflammatory bowel disease, where it can synergize with IFN-γ to regulate multiple tight-junction (TJ) proteins, including myosin light chain kinase, occludin, and ZO-1, as well as claudin-1 and claudin-2 (106–109), which are also regulated by IL-17. Interestingly, neither TNF-α nor IL-17 were changed in response to infection in our model. In accord with our observations, decreased TNF-α in children with malaria and bacteremia relative to children with malaria alone has been observed previously (2). In our model, TNF-α levels may be affected by elevated levels of Mcpt1 and Mcpt4, since both chymases not only degrade proteins of the basement membrane and extracellular matrix (110, 111), but they can also degrade cytokines such as TNF-α (35) and IL-33 (41). The lack of IL-1β and TNF-α, both typically elevated in malarial disease (112, 113), may also be due to downregulation by IL-10 (Fig. 6B), which increases with rising parasitemia (Fig. 1A). In addition to these effects, IL-10 can provide a protective role against TJ barrier disturbance, since IL-10 deficiency has been associated with increased intestinal permeability (114, 115) and mislocalization of ZO-1 and claudin-1 away from TJs, perhaps by the action of increased proinflammatory TNF-α, IL-1, and IL-6 (116). The administration of IL-10 can also prevent IFN-γ-induced barrier dysfunction (117), but the effects of IL-10 can vary based on context. For example, IL-10 can enhance IgE-mediated MC-dependent barrier dysfunction during food allergy (118).

MC proteases have known roles in intestinal permeability and have been studied through the generation of mouse strains deficient in the chymases Mcpt1, Mcpt2, Mcpt4, and Mcpt5 (119). Mcpt1 was the first chymase described to be involved in expulsion of Trichinella spiralis from the intestine of infected mice (120). The regulation of gut barrier function, including permeability and epithelial migration, by the human chymase homologue Mcpt4, was subsequently reported (42). In general, it has been shown that the activation of protease-activated receptor 2 (PAR-2) by human chymase induces metalloprotease-2 (MMP-2) expression that is associated with reduced claudin-5 and epithelial barrier dysfunction (121). In our model, both Mcpt1 and Mcpt4 were observed in circulation in parasite-infected mice, but Mcpt4 increased first (days 4 and 8 p.i.; Fig. 3C), while Mcpt1 (Fig. 3D) was detected with the second peak of ileal MCs (day 8 p.i.; Fig. 3A), suggesting that these chymases are induced by different mechanisms and play different roles in gut regulation. Indeed, it has been shown that Mcpt1 and Mcpt4 have different preferences in their sequence target peptides (122).

In conclusion, P. yoelii *yoelii* 17XNL infection induces increased intestinal permeability and significant elevation of bacterial 16S copies in the blood preceded by MC proliferation in the ileum, a marked Th2-type allergic response, and increased MC mediators in circulation, suggesting that activated MCs and/or their products may promote and regulate not only the intestinal cytokine responses but also patterns of host immunity to malaria infection. Thus, our mouse model provides an experimental setting for the study intestinal immune responses and intestinal barrier function during nonlethal malaria-associated bacteremia. Studies are ongoing to confirm the contribution of activated MC factors to the coordination of Th1 and Th2 immunity as well as changes to the integrity of the intestinal barrier and patterns of bacteremia in malaria.

## MATERIALS AND METHODS

### Mice.

Female, 8-week-old C57BL/6J mice (000664) were obtained from Jackson Laboratory and housed in ventilated microisolator caging and provided food and water *ad libitum*. All procedures were approved by the Institutional Animal Care and Use Committee of the University of Idaho.

### Mouse infection and monitoring.

Mice were injected with 150 μl of P. yoelii *yoelii* 17XNL-infected red blood cells (1 × 10^6^ parasites) (*n* = 90) or uninfected red blood cells (*n* = 26) at day 0 by intraperitoneal (i.p.) administration. Daily parasitemia was recorded from thin blood films stained with Giemsa beginning on day 2 p.i. Mice were monitored daily for weight loss and reduced activity. Blood samples were collected by cardiac puncture for complete blood counts (CBCs) and determination of bacterial 16S DNA copies. Plasma samples were collected for analysis of IgE, cytokines, chemokines, and Mcpt1 and Mcpt4; these samples were frozen at −80°C until analysis. Ileum tissue was collected and saved in formalin for immunohistochemistry studies.

### CBCs.

Whole blood was collected via cardiac puncture in EDTA tubes at the time of necropsy. These samples were mixed gently and shipped overnight to the Comparative Pathology Lab at the University of California, Davis. Samples were analyzed within 24 h with a Hemavet 950FS automated hematology system.

### Creation of 16S bacterial DNA plasmid standard.

To create a standard curve for quantification by qPCR, genomic DNA was isolated from Escherichia coli using a DNeasy blood and tissue kit (Qiagen) according to the manufacturer’s protocol. Genomic DNA was then amplified using primers for eubacterial 16S ribosomal DNA (forward 5′-ACTCCTACGGGAGGCAGCAGT-3′, reverse 5′-ATTACCGCGGCTGCTGGC-3′. The 197-bp product was cloned using the TOPO-TA cloning kit (Invitrogen) following the manufacturer’s protocol. After transformation and overnight growth on agar plates, colonies were selected and grown in LB broth (Thermo Fischer). Plasmid DNA was isolated using the QIAprep Spin miniprep kit (Qiagen), and the insert was confirmed by restriction digest and Sanger sequencing. The plasmid was diluted to 109 copies/μl, and a 10-fold dilution series (109 copies/μl to 101 copies/μl) was created and used as a standard curve. The calculated limit of detection of this assay is 10 copies/μl, determined as the lowest concentration at which no more than 5% failed reactions occur (123).

### Extraction of DNA from blood for 16S quantitative PCR (qPCR).

Whole blood was collected in EDTA tubes via cardiac puncture at the time of necropsy, flash frozen in liquid nitrogen, and stored at −80°C. Total DNA was isolated using DNeasy blood and tissue kits (Qiagen) according to the manufacturer’s protocol.

### DNA.

DNA was diluted to 4 ng/μl, and reaction mixtures of 12 μl containing 6 μl Maxima SYBR green/ROX qPCR master mix (2×) (Bio-Rad), 0.5 μl of the forward and reverse 16S primer at 10 μM, 2.5 μl of water, and 2 μl of DNA (normalized to 4 ng/μl) were analyzed in triplicate to confirm uniform amplification using the following cycling conditions: 50°C for 2 min, 95°C for 10 min, and 40 cycles of 95°C for 15 s and 60°C for 1 min.

### *In vivo* intestinal permeability.

Mice were fasted for 4 h before oral gavage with 50 mg/100 g body weight of 4-kDa fluorescein isothiocyanate dextran (FITC) in sterile phosphate-buffered saline (PBS). After 3 h, blood was collected and plasma was separated and diluted with PBS (pH 7.4, 1:2 vol/vol). Standard curves were obtained by serial dilution of FITC-dextran in normal mouse plasma diluted with PBS (1:2 vol/vol). Plasma fluorescence was analyzed using a microplate reader (Molecular Devices LLC, San Jose, CA) at excitation/emission wavelengths of 490/520 nm.

### *Ex vivo* intestinal sac assay.

Intestinal permeability was assessed as described in Mateer et al. (40) with slight modifications. Mice were fasted for 4 h prior to euthanasia. At necropsy, ileal segments were isolated and gently flushed with 1× PBS to remove any remaining contents. The distal end was ligated with a nylon suture and filled with 1 mg/ml of 4-kDa FITC-dextran (Sigma-Aldrich) dissolved in phenol-free Dulbecco’s modified Eagle medium (DMEM) (Thermo Fisher). The proximal end of the segment was ligated and placed into a 50-ml conical tube with 20 ml of 37°C DMEM and incubated in a 37°C water bath for 120 min. Samples of medium were removed at 0, 30, 60, 90, and 120 min, and the amount of translocated FITC-dextran was quantified using a fluorescent plate reader. Apparent permeability was calculated as described in Mateer et al. (40).

### Cytokines and chemokines in plasma samples.

Concentrations of plasma cytokines (IL-1β, IL-3, IL-4, IL-10, IL-6, IL-2, IL-9, IL-12p70, IL-12p40, IL-13, IFN-γ, TNF-α, MCP-1, MIP-1α, MIP-1β, RANTES, eotaxin, and KC) were determined using a Bio-Plex Pro Luminex assay. Briefly, 25 μl of serum was incubated with fluorescently labeled capture antibody-coated beads in a 96-well filter-bottomed plate on a plate shaker overnight at 4°C. After incubation, the sample-bead mix was removed, and the plate was washed twice using a vacuum manifold. Biotinylated detection antibodies were then added and incubated for 1 h at room temperature with shaking. The reaction mixture was detected by the addition of streptavidin-phycoerythrin and incubated on a plate shaker at room temperature for 30 min. Following a repeat of the washing step, beads were resuspended in sheath fluid for 5 min on the plate shaker. Plates were read on a Bio-Plex 200 system (Bio-Rad Laboratories, Hercules, CA, USA) and analyzed using Bio-Plex Manager software (Bio-Rad Laboratories) with a five-parameter model used to calculate final concentrations and values (expressed in pg/ml). Reference samples were run on each plate to determine assay consistency, and all samples were run in blinded experimental groups.

### ELISAs.

Levels of circulating IgE (eBioscience; Thermo Fisher Scientific, Inc.), Mcpt1 (eBioscience), Mcpt4 (Aviva Systems Biology), and IL-18 (BMS618-3; eBioscience) were determined in plasma samples using commercial enzyme-linked immunosorbent assay (ELISA) kits according to the manufacturer’s instructions and a microplate reader (Molecular Devices LLC, San Jose, CA).

### Ileum histochemistry.

Ileum samples collected at necropsy were formalin-fixed and embedded in paraffin. From these tissue blocks, 5-μm sections were cut, deparaffinized in xylene, rehydrated in graded solutions of alcohol, and subjected to enzyme histochemical staining to identify naphthol AS-D chloroacetate esterase (NASDCE) activity (ref. 91C-1KT; Sigma-Aldrich), which detects chymases in MC secretory granules (124). For each mouse examined, MCs were enumerated in 30 to 50 high-power fields (HPF).

### Statistical analysis.

Bacterial 16S DNA copies per μl of blood, number of MCs per HPF in ileum tissue, IgE, and cytokine concentration in plasma were compared between different time point groups using the Kruskal-Wallis test followed by Dunn’s multiple-comparison test of each time point with the control group. *P* values of <0.05 were considered significant.

### Ethics statement.

All experiments were performed with the approval of the Institutional Animal Care and Use Committee of the University of Idaho (protocol number 2017-20).

## Supplementary Material

Supplemental file 1
