# Supplementary material for: Nonlethal Plasmodium yoelii Infection Drives Complex Patterns of Th2-Type Host Immunity and Mast Cell-Dependent Bacteremia
Source: Infect Immun. 2020 Nov 16;88(12):e00427-20. doi: 10.1128/IAI.00427-20 (PMC7671899; doi:10.1128/IAI.00427-20)
Supplement: Supplemental file 1 [file IAI.00427-20-s0001.pdf]

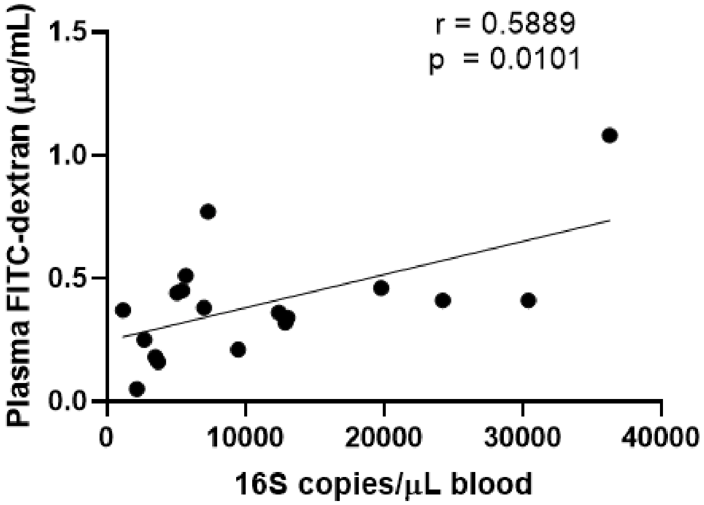

Figure S1. Correlation between plasma FITC-dextran and bacterial 16S copy number. The x-axis represents bacterial 16S copy numbers per µL of blood, and y-axis the concentration of FITC-dextran in plasma 3 hours following oral gavage. Pearson r = 0.0.5889, p = 0.0101. Each dot represents one mouse.
